# Use of antiviral drugs and incidence of Parkinson’s disease in Taiwan

**DOI:** 10.1371/journal.pone.0302383

**Published:** 2024-05-07

**Authors:** Han-Wei Zhang, Jun Xie, Hsiao-Ching Peng, Yu-Shan Lin, Ji-Quan Song

**Affiliations:** 1 Department of Dermatology, Zhongnan Hospital of Wuhan University, Wuhan, China; 2 Program for Aging, China Medical University, Taichung, Taiwan; 3 Department of Electrical and Computer Engineering, Institute of Electrical Control Engineering, National Yang Ming Chiao Tung University, Hsinchu, Taiwan; 4 MetaTrial Research Center, Biomedica Corporation, New Taipei, Taiwan; Lerner Research Institute - Cleveland Clinic, UNITED STATES

## Abstract

Patients infected with herpes zoster might be at risk for Parkinson’s disease (PD). However, antiviral drugs may impede viral deoxyribonucleic acid (DNA) synthesis. This study aimed to determine whether the currently observed association between herpes zoster and PD is consistent with previous findings, and whether antiviral drug use is associated with PD. This retrospective cohort study used the Longitudinal Generation Tracking Database. We included patients aged 40 years and above and applied propensity score matching at 1:1 ratio for study comparability. PD risk was evaluated using Cox proportional hazards regression methods. A total of 234,730 people were analyzed. The adjusted hazard ratio (aHR) for PD in patients with herpes zoster was 1.05. Furthermore, the overall incidence of PD was lower in those treated with antiviral drugs than in the untreated ones (3.17 vs. 3.76 per 1,000 person-years); the aHR was 0.84. After stratifying for sex or age, a similar result was observed. In conclusion, herpes zoster may increase the risk of PD, particularly among females, but receiving antiviral treatment reduces the risk by 16%. Therefore, using antiviral drugs may help prevent PD. However, additional research is required to determine the underlying mechanism(s).

## Introduction

Parkinson’s disease (PD) is a chronic and progressive neurodegenerative disorder. It rarely occurs before the age of 40 and then increases progressively with age. It typically affects those over the age of 60, along with stroke and dementia. In fact, it is classified as one of the three main diseases affecting geriatric health. PD is characterized by the degeneration of the substantia nigra in the midbrain and the appearance of intracellular Lewy bodies. Owing to dopamine decrease, the brain loses essential compounds that help regulate muscle activity, thereby inhibiting the nerve conduction pathway from the basal ganglia to the motor cortex and resulting in aberrant motor function. Hands and feet tremor, rigidity, sluggish movement, and unstable posture are the primary symptoms.

Human alpha herpesviruses may contribute to the etiology of neurodegenerative diseases [1, 2]. These chronic neurotropic viruses generally infect the human host’s peripheral nervous system. After a time of dormancy, they may revive or induce recurring sickness inside neuronal sensory cells [3]. Herpes simplex virus (HSV) 1 and 2, as well as varicella zoster virus (VZV), belong to the alpha herpesvirus subfamily. Furthermore, the normal and neuropathogenic activities of α-synuclein may be molecularly distinct, leading to neurodegeneration [1]. PD is classified as a synuclein lesion caused by the α-synuclein development of aggregates in the shape of Lewy bodies [4]. According to a review, herpes zoster may be related to PD because of the inflammation and immunological alterations involved in both disorders [5]. Furthermore, a recent study suggests that infection may lead to a sequence of imbalances in the gut microbiota, involvement of glial tissue, inflammation of the nervous system, and buildup of α-synuclein [6]. These variables have the potential to develop and worsen PD. However, recent a case-control study has contradictory findings [2]. Consequently, while the majority of research indicates herpes zoster as a potential contributing risk factor in the development of PD [5–8], the necessity for conducting cohort study remains to elucidate the intricacies of this relationship. Additionally, anti-infective drugs have been proposed as PD therapeutic targets [9]. Several antiviral drugs, including acyclovir, valacyclovir, and famciclovir, have shown efficacy against VZV. The primary methods by which these medications exert their antiviral effects include inhibiting the viral deoxyribonucleic acid (DNA) polymerase and incorporating themselves into the freshly manufactured viral DNA, thereby impeding the virus’s ability to complete the replication process. Consequently, we hypothesized that antiviral drug use can potentially reduce the occurrence of aberrant protein aggregation in PD [1], thus exerting a preventive effect. This research mainly aimed to undertake a retrospective cohort study to better comprehend this notion. Additionally, we reassessed the association between PD and the herpes zoster and investigated the potential linkage between antiviral drug use and PD onset at different stratifications.

## Materials and methods

### Data sources

Information that includes the claims data of two million randomly sampled beneficiaries in 2000–2018 was procured from the Longitudinal Generation Tracking Database 2005 (LGTD 2005) within the National Health Insurance Research Database (NHIRD) in Taiwan. The data were deidentified and anonymized before being taken from the database. Thus, the China Medical University Hospital Clinical Trial Center (CMUH111-REC2-109[CR-1]) approved this study. The Research Ethics Committee waived the right to obtain informed consent from the participants. All results produced or analyzed as part of this study are incorporated in this article.

Established in 1996 in Taiwan, the NHRD contains all the medical histories and insurance claims data obtained from the National Health Institute (NHI) database, which encompasses the healthcare information of 99% of population in Taiwan, covered by a universal health insurance program [10]. The NHIRD, which holds data on the outcomes of real-world practice, has been mostly recognized for its significance and clinical influence beyond the results obtained from clinical trials conducted at multiple centers that provide support for developing guidelines for disease management in clinical practice [11]. The NHI has implemented stringent guidelines for record keeping to detect fraud and unethical behavior in medical practice. In addition, the NHI can impose fines that are 100 times the amount of money spent on healthcare. Therefore, the NHIRD provides trustworthy and accurate healthcare information for investigating real-world evidence, according to Big Health Data analytics. Using the International Classification of Disease, Ninth Revision, Clinical Modification (ICD-9-CM), the database documents the health status of every individual.

### Study design and study population

**Fig 1:**
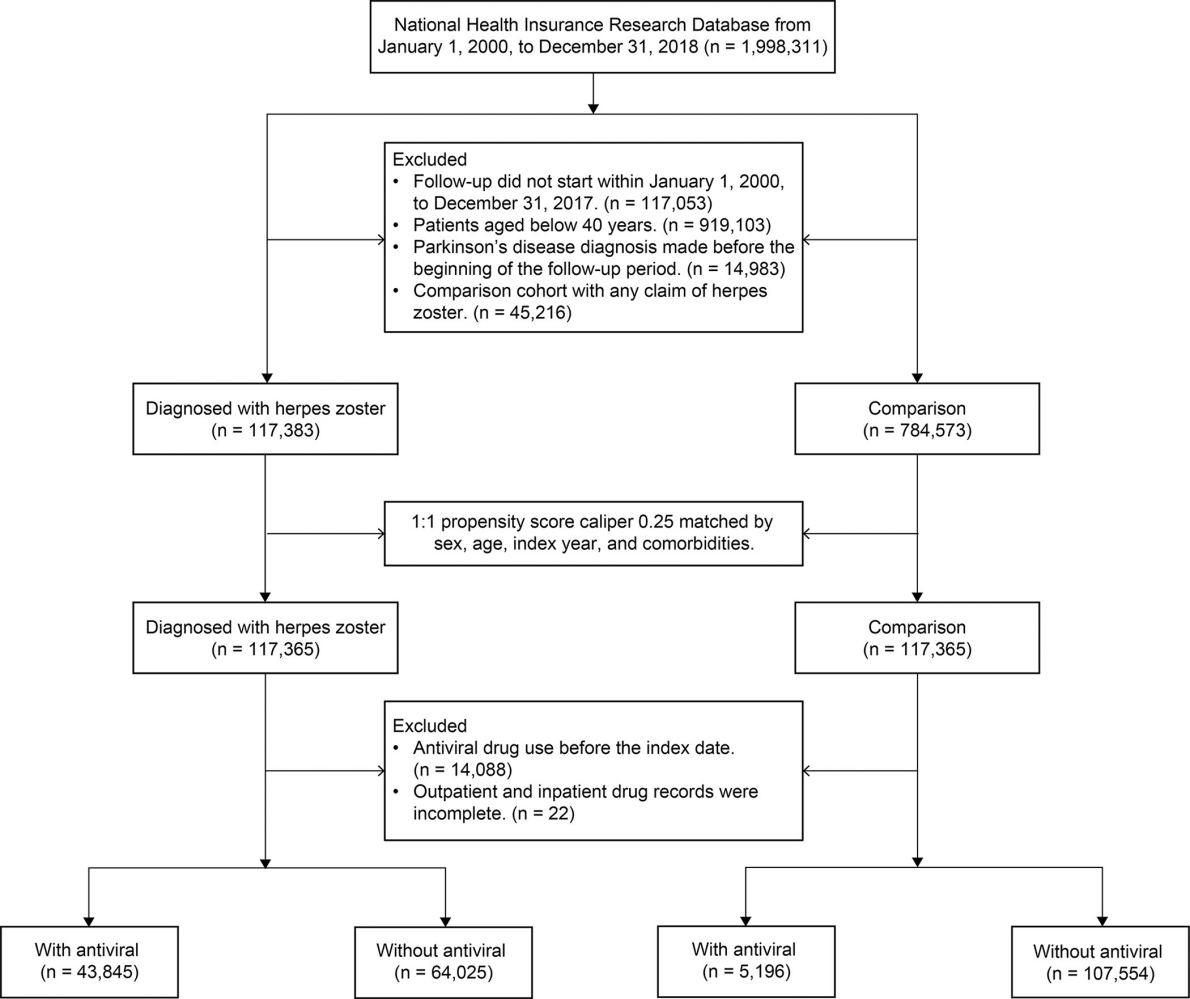
Flowchart of study participant selection.

This retrospective cohort study included inpatient and outpatient claims data from LGTD recorded between January 1, 2000, and December 31, 2018. However, to guarantee the validity and quality of illness definition, we further established the following criteria. (1) To provide an adequate duration for observing the outcome in this investigation, we only included patients whose index dates fell within the time frame of 2000–2017. Consequently, a minimum follow-up duration of 1 year was observed in the analysis. (2) We excluded individuals aged below 40 years. (3) To ensure new-onset PD, we excluded individuals diagnosed with PD before the beginning of the follow-up period. (4) To improve the rigor of research, we excluded individuals who had any claim of herpes zoster from the comparison cohort. Ultimately, we analyzed 234,730 individuals. Furthermore, to conduct a more comprehensive investigation of the potential link between antiviral drug use and PD, we omitted any instances of prior antiviral drug usage before the specified index date. Those who had incomplete drug data were also excluded from the study. Fig 1 shows the selection flow of the study population.

### Selection of exposure

From the included population, we identified individuals who were newly diagnosed with herpes zoster (ICD-9-CM code 053) between 2000 and 2013 (herpes zoster cohort). For those who reported at least two outpatient visits or one hospitalization, we determined those who received antiviral treatment [Anatomical Therapeutic Chemical code D06BB03, J05AB01, S01AD03, J05AB11 and J05AB09] and those who did not. Conversely, the comparison cohort comprised individuals from the same dataset but without herpes zoster. This cohort was established using propensity score matching (PSM) at 1:1 ratio. The following variables were included in the multiple logistic regression to derive propensity scores: sex, age, index year, and comorbidities (hypertension, diabetes mellitus, coronary artery disease, hyperlipidemia, cerebrovascular diseases, head injury, depression, congestive heart failure, atrial fibrillation, cancer, liver disease, chronic infection, autoimmune disease, malnutrition, dementia, smoking-related diagnoses, pesticide poisoning, migraine with aura, and anemia). Eventually, 234,730 individuals were recruited for the two cohorts, and each cohort had 117,365 participants. For all subsequent analyses, the very first date of herpes zoster diagnosis was defined as the index date and the beginning of the follow-up period.

### Selection of outcomes

In this study, the primary outcome measure was new cases of PD diagnosis during the follow-up period. The diagnosis was based on the ICD-9-CM code 332 and at least two outpatient visits or one hospitalization to prevent miscoding patients. The very first hospitalization or an outpatient clinic visitation date, with PD diagnosis, was defined as the date of diagnosis as well as the date of recently diagnosed PD for all successive analyses. We used PD diagnosis, withdrawal from the NHI program, or December 31, 2018, whichever occurred first, as the study endpoint.

### Potential confounding factors

With the advancement of epidemiological research methods, the study on PD etiologies has been expanded, but its specific cause remains unclear. Several epidemiological studies have demonstrated that PD prevalence and incidence increase with age [12–15], suggesting that age is closely associated with PD development; however, this association does not prove that age is the cause of PD. Rather, age could be a significant risk factor. However, sex, urbanization, and socioeconomic status may also be risk factors for PD [12, 14, 16]. In addition, other acquired factors, such as environmental exposure (e.g., pesticide exposure, heavy metal exposure, and air pollution), as well as the accumulation of risk factors in the body, may individually or jointly lead to the formation of diseases. Exposure to highly active specific pesticides or other factors related to agriculture may be causally linked to PD [17–19]. In addition, the cumulative effect of toxic effects may result in an accelerated course of a progressive disease [19]. Despite differences in study size, most studies provided similar risk estimates that pesticide exposure is positively associated with PD [20]. Moreover, some cohort studies have shown that cerebrovascular risk factors (prior stroke, atrial fibrillation, coronary heart disease, hyperlipidemia, heart failure, etc.) [21, 22] and migraine [23] are associated with PD and that the autoimmune disease-associated genetic variants in CLEC16A are linked to PD susceptibility in Han Chinese [24]. Systematic reviews and meta-analyses also reported that cancer [25], hypertension [26], diabetes [27], depression [28], infection [29, 30], malnutrition [31], head injury [32], anemia [33], and stroke [34] are associated with increased PD risk. However, smoking is inversely associated with PD [35]. Researchers speculate that nicotine in tobacco products increases the survival rate of dopamine neurons and reduce metabolic toxicants in the body [36, 37].

In this study, comorbidity was based on ICD-9-CM codes in the LGTD. Patients were recognized and described according to the diagnosis history acquired from a minimum of two outpatient visits or at least one hospitalization before the date of newly diagnosed PD. Comorbidities that were considered in the analyses were as follows: hypertension (401–405), diabetes mellitus (250), coronary artery disease (410–414), hyperlipidemia (272), cerebrovascular diseases (430–438), head injury (310.2, 800, 801, 803, 804, 850, 851, 853, 854, and 959.01), depression (296.2, 296.3, 296.82, 300.4, and 311), congestive heart failure (428), atrial fibrillation (427.31 and 427.3), cancer (140–165, 170–176, and 179–208), liver disease (571 and 070), chronic infection (042, 010–018, and 090–099), autoimmune disease (279), malnutrition (260–269), dementia (290, 294.1, and 331.0), smoking-related diagnoses (305.1, 491.0, 491.2, 492.8, 496, 523.6, 989.84, V15.82, and 649.0), pesticide poisoning (989.1–989.4), migraine with aura (346.0), and anemia (285.9). Of note, although some confounding factors were inconsistent in their association with PD, they were still considered in this study to avoid potential bias caused by these confounding factors.

### Statistical analysis

The descriptive data are presented as mean ± standard deviation (SD) and frequencies with percentages (%) for continuous and categorical variables, respectively. The chi-squared test and Student’s *t*-test were applied to evaluate the differences in demographic characteristics and comorbidity distribution between the matched control and herpes zoster cohorts to elucidate the relationship of PD development with the presence of herpes zoster and the use/nonuse of antiviral drugs. The PD risk between the cohorts, expressed as HRs with 95% CIs, was evaluated using the Cox proportional hazards regression model. We estimated the independent effects in incident PD by adjusting for age, sex, and unbalanced potential confounding factor. A stratified analysis was conducted to examine whether the effects differed in sex or age. The presence of a trend in the cumulative utilization of antiviral drugs and the occurrence of PD was evaluated using the Cochran–Armitage trend test. Furthermore, the likelihood of people developing PD throughout the course of the follow-up period was determined using the Kaplan–Meier method. Any observed differences were assessed using the log-rank test. The fitness of the model was also evaluated using the Schoenfeld residual test to examine the proportionality assumption between herpes zoster and the antiviral drug therapy (S1–S3 Figs). All analyses were conducted using the SAS version 9.4 software (SAS Institute, Inc., Cary, NC) and the R version 4.3.1 software (R Core Team, Vienna, Austria), with the use of the survival package in R. The statistical tests were two-sided, and a *P* value of 0.05 or less was considered statistically significant.

## Results

### Study population characteristics

Table 1 lists the demographic data and confounding factors of both cohorts after PSM. The mean age of each cohort, which included 117,365 individuals, was 61.40 ± 12.09 years. The analysis of matched patients revealed a satisfactory distribution between the herpes zoster and comparison cohorts for the parameters of some of the comorbidities, including hyperlipidemia, congestive heart failure, cancer, malnutrition, smoking-related diagnoses, and pesticide poisoning. The mean duration of follow-up for individuals with PD was 7.49 years in the herpes zoster cohort and 7.25 years in the comparison cohort. However, both cohorts had a high likelihood of originating from metropolitan regions or families with moderate monthly earnings.

**Table 1:** Characteristics of the herpes zoster and comparison cohorts.

**Characteristics**	**n (%)**		***P* value**
	**Comparison cohort** **(n = 117,365)**	**Herpes zoster cohort** **(n = 117,365)**	
**Age, years**			>0.999
40–64	72,723 (61.96)	72,723 (61.96)	
≥65	44,642 (38.04)	44,642 (38.04)	
Mean ± SD	61.40 ± 12.09	61.40 ± 12.09	>0.999
**Sex**			>0.999
Male	52,952 (45.12)	52,952 (45.12)	
**Urbanization level** ^*a*^			<0.001
1 (highest)	56,223 (47.90)	57,641 (49.11)	
2	37,095 (31.61)	36,487 (31.09)	
3	7,377 (6.29)	7,369 (6.28)	
4 (lowest)	867 (0.74)	777 (0.66)	
Unknown	15,803 (13.46)	15,091 (12.86)	
**Insurance amount** ^***b***^ **, NT$**			<0.001
Financially dependent	159 (0.14)	80 (0.07)	
1–19,999	37,340 (31.82)	30,370 (25.88)	
20,000–39,999	60,049 (51.16)	64,215 (54.71)	
≥40,000	19,817 (16.88)	22,700 (19.34)	
**Comorbidity** ^***c***^			
Hypertension	75,446 (64.28)	73,875 (62.94)	<0.001
Diabetes mellitus	43,436 (37.01)	42,833 (36.50)	0.010
Coronary artery disease	43,777 (37.30)	43,032 (36.67)	0.001
Hyperlipidemia	63,946 (54.48)	63,820 (54.38)	0.602
Cerebrovascular diseases	30,323 (25.84)	29,438 (25.08)	<0.001
Head injury	13,326 (11.35)	14,035 (11.96)	<0.001
Depression	13,955 (11.89)	15,135 (12.90)	<0.001
Congestive heart failure	15,640 (13.33)	15,571 (13.27)	0.675
Atrial fibrillation	7,532 (6.42)	7,273 (6.20)	0.028
Cancer	16,793 (14.31)	16,536 (14.09)	0.129
Liver disease	38,624 (32.91)	39,340 (33.52)	0.002
Chronic infection	5,918 (5.04)	6,229 (5.31)	0.004
Autoimmune disease	1,047 (0.89)	1,666 (1.42)	<0.001
Malnutrition	2,097 (1.79)	2,079 (1.77)	0.779
Dementia	10,433 (8.89)	9,761 (8.32)	<0.001
Smoking-related diagnoses	28,750 (24.50)	28,559 (24.33)	0.359
Pesticide poisoning	219 (0.19)	241 (0.21)	0.305
Migraine with aura	847 (0.72)	1,195 (1.02)	<0.001
Anemia	17,325 (14.76)	17,849 (15.21)	0.002
**Follow-up time, years**			<0.001
Mean ± SD	7.25 ± 4.84	7.49 ± 4.85	

SD, standard deviation.

Values are expressed as means ± SD or number (percentage).

^*a*^Urbanization level was defined at the beginning of the follow-up period.

^*b*^Insurance amount was measured as the average value during the follow-up period.

^*c*^Comorbidity was defined before the survival date.

### Associations between herpes zoster and PD

Table 2 lists the associations of PD risks between the two cohorts. The herpes zoster cohort and comparison cohort (control) were followed up for 879,225 and 851,094 person-years, respectively. The herpes zoster cohort had a higher rate of overall PD incidence (3.70 vs. 3.50 per 1,000 person-years) and a significantly higher hazard ratio (HR) for PD (1.06; 95% confidence interval [CI], 1.01–1.11) than the comparison cohort. However, when controlling for potential confounding factors, the HR for PD did not attain statistical significance (1.05; 95% CI, 1.00–1.10; *P* = 0.057). Regarding cumulative PD incidence, the herpes zoster group still achieved a higher rate than the comparison group (log-rank test, *P* = 0.027; S4 Fig).

**Table 2:** Stratified analysis of PD incidence and HRs for PD in the herpes zoster and comparison cohorts.

Population	Study group	PD	PY	Incidence Rate^*a*^	Crude HR (95% CI)	*P* value	Adjusted HR^*b*^ (95% CI)	*P* value
**Total**	Comparison (n = 117,365)	2,977	851,094	3.50	1 (reference)	-	1 (reference)	-
	Herpes zoster (n = 117,365)	3,257	879,225	3.70	1.06 (1.01, 1.11)	0.027	1.05 (1.00, 1.10)	0.057
**Female**	Comparison (n = 64,413)	1,557	484.296	3.21	1 (reference)	-	1 (reference)	-
	Herpes zoster (n = 64,413)	1,698	489,107	3.47	1.08 (1.01, 1.16)	0.027	1.09 (1.01, 1.16)	0.020
**Male**	Comparison (n = 52,952)	1,420	366,798	3.87	1 (reference)	-	1 (reference)	-
	Herpes zoster (n = 52,952)	1,559	390,118	4.00	1.03 (0.96, 1.11)	0.422	1.01 (0.94, 1.09)	0.762
**Age 40–64 years**	Comparison (n = 72,723)	829	585,779	1.42	1 (reference)	-	1 (reference)	-
	Herpes zoster (n = 72,723)	879	594,195	1.48	1.04 (0.95, 1.14)	0.411	1.08 (0.98, 1.19)	0.106
**Age ≥65 years**	Comparison (n = 44,642)	2,148	265,315	8.10	1 (reference)	-	1 (reference)	-
	Herpes zoster (n = 44,642)	2,378	285,030	8.34	1.03 (0.97, 1.09)	0.363	1.04 (0.98, 1.10)	0.184

PD, Parkinson’s disease; CI, confidence interval; HR, hazard ratio; PY, person-years.

^*a*^-years.

^*b*^Cox regression models were adjusted for age, sex, urbanization level, insurance amount, and comorbidities such as hypertension, diabetes mellitus, coronary artery disease, cerebrovascular diseases, head injury, depression, atrial fibrillation, liver disease, chronic infection, autoimmune disease, dementia, migraine with aura, and anemia.

We further stratified the population according to sex (female vs. male) and age (40–64 years vs. ≥65 years) to analyze the associations between herpes zoster and PD occurrence. Compared with that in the comparison group, the HR of PD was higher in females with herpes zoster (1.09 [95% CI, 1.01–1.16]; *P* = 0.020) than in their male counterpart (1.01 [95% CI, 0.94–1.09]; *P* = 0.762), and in patients with herpes zoster aged 40–64 years (1.08 [95% CI, 0.98–1.19]; *P* = 0.106) than in those aged ≥65 years (1.04 [95% CI, 0.98–1.10]; *P* = 0.184). However, statistical significance was only observed in the female group. Therefore, the possibility of having a new diagnosis of PD may be higher by 9% in females with herpes zoster than in those without.

### Associations between antiviral drug use and PD

**Fig 2:**
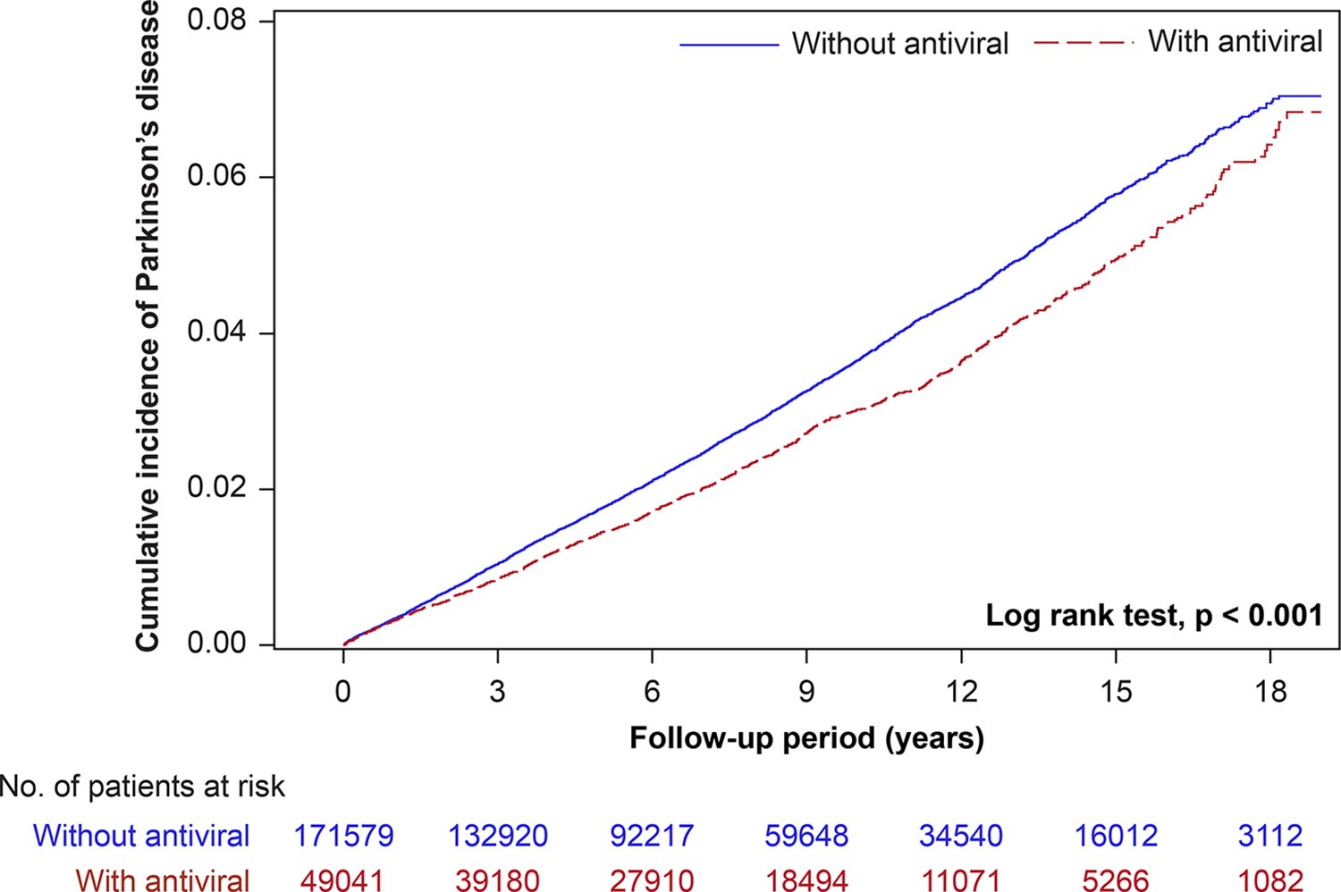
Cumulative incidence curves of PD in individuals treated with and without antiviral drugs.

Table 3 compares PD risks in patients treated with and without antivirals. We identified 49,041 individuals who received antiviral treatment after the index date and 171,579 who never received it; they were observed for 381,369 and 1,269,067 person-years, respectively. The overall PD incidence rate was lower in the antiviral treatment group than in the nontreatment group (3.17 vs. 3.76 per 1,000 person-years). After accounting for potential confounding factors, the HR for PD was considerably reduced in the treatment group (0.84; 95% CI, 0.79–0.90). Thus, patients with herpes zoster taking antiviral drugs had a lower chance of being diagnosed with PD (16%) than those who did not receive such treatment. We further subgrouped the patients according to the cumulative number of days of antiviral treatment (1–6, 7–13, and ≥14 days). The HR of PD was 0.87 (95% CI, 0.80–0.94; *P* < 0.001) for the 1–6 days of treatment, 0.85 (95% CI, 0.76–0.96; *P* = 0.006) for the 7–13 days, and 0.78 (95% CI, 0.68–0.89; *P* < 0.001) for the ≥14 days, compared with that in the nontreatment group. The duration of cumulative antiviral drug use clearly correlated with the likelihood of acquiring PD, as shown by a statistically significant downward trend (*P* trend = 0.022). The Kaplan–Meier analysis revealed that individuals undergoing antiviral treatment had a reduced cumulative incidence of PD (log-rank test, *P* < 0.001; Fig 2).

**Table 3:** PD risk in patients treated with and without antiviral drugs.

Antiviral treatment	PD	PY	Incidence Rate^*a*^	Adjusted HR^*b*^ (95% CI)	*P* value
Without (n = 171,579)	4,767	1,269,067	3.76	1 (reference)	-
With (n = 49,041)	1,208	381,369	3.17	0.84 (0.79, 0.90)	<0.001
**Cumulative days**					
Non-used (n = 171,579)	4,767	1,269,067	3.76	1 (reference)	-
1–6 days (n = 28,364)	666	217,249	3.07	0.87 (0.80, 0.94)	<0.001
7–13 days (n = 12,497)	314	95,615	3.28	0.85 (0.76, 0.96)	0.006
≥14 days (n = 8,180)	228	68,504	3.33	0.78 (0.68, 0.89)	<0.001
***P* for trend**					0.022

PD, Parkinson’s disease; CI, confidence interval; HR, hazard ratio; PY, person-years.

^*a*^per 1,000 person-years.

^*b*^Cox regression models were adjusted for age, sex, urbanization level, insurance amount, and comorbidities such as hypertension, diabetes mellitus, coronary artery disease, cerebrovascular diseases, head injury, depression, atrial fibrillation, liver disease, chronic infection, autoimmune disease, dementia, migraine with aura, and anemia.

To assess the specific differences of association and reveal any unexpected hidden relationships between antiviral drug use and PD development, we conducted stratified analyses to compute causal effects within the underlying strata of sex or age (Table 4). The findings align with the patterns seen in the broader population. The incidence of PD was considerably lower among those who used antiviral treatments, irrespective of sex and age (40–64 and 65 years), than that among those who did not use. The risk reduction percentages were 17%, 15%, 15%, and 16% for female, male, middle-aged (40–64 years), and senior (65 years) users, respectively. In addition, the cumulative days of using antiviral drugs also demonstrated the same statistical effect on PD risk. Notably, statistical significance was only observed in the female or senior population (*P* trend < 0.05). Moreover, given that the incidence of herpes zoster reportedly correlates with PD development, we investigated the use of antiviral drugs among those afflicted with herpes zoster (S1 Table). In the herpes zoster group, antiviral drug treatment was inversely associated with PD development, consistent with the broader population. Additionally, the cumulative PD incidence rate was lower in the antiviral treatment group than in the nontreatment group (log-rank test, *P* < 0.001; S5 Fig).

**Table 4:** Stratified analysis of PD risk in patients treated with and without antiviral drugs.

Population	Antiviral treatment	PD	PY	Incidence Rate^*a*^	Adjusted HR^*b*^ (95% CI)	*P* value
**Female**	Without (n = 92,652)	2,482	704,370	3.52	1 (reference)	-
	With (n = 27,452)	632	219,355	2.88	0.83 (0.76, 0.91)	<0.001
	**Cumulative days**					
	Nonuse (n = 92,652)	2,482	704,370	3.52	1 (reference)	-
	1–6 days (n = 16,044)	364	126,411	2.88	0.86 (0.77, 0.97)	0.010
	7–13 days (n = 7,023)	156	54,755	2.85	0.81 (0.69, 0.95)	0.010
	≥14 days (n = 4,385)	112	38,189	2.93	0.77 (0.64, 0.94)	0.008
	***P* for trend**					0.008
**Male**	Without (n = 78,927)	2,285	564,698	4.05	1 (reference)	-
	With (n = 21,589)	576	162,013	3.56	0.85 (0.78, 0.93)	<0.001
	**Cumulative days**					
	Nonuse (n = 78,927)	2,285	564,698	4.05	1 (reference)	-
	1–6 days (n = 12,320)	302	90,837	3.32	0.86 (0.77, 0.98)	0.018
	7–13 days (n = 5,474)	158	40,861	3.87	0.89 (0.76, 1.05)	0.164
	≥14 days (n = 3,795)	116	30,315	3.83	0.77 (0.64, 0.93)	0.007
	***P* for trend**					0.607
**Age 40–64 years**	Without (n = 10,694)	1,281	861,929	1.49	1 (reference)	-
	With (n = 30,250)	352	260,606	1.35	0.85 (0.75, 0.96)	0.007
	**Cumulative days**					
	Nonuse (n = 105,694)	1,281	861,929	1.49	1 (reference)	-
	1–6 days (n = 18,079)	191	152,477	1.25	0.83 (0.71, 0.97)	0.017
	7–13 days (n = 7,530)	89	63,951	1.39	0.89 (0.72, 1.10)	0.283
	≥14 days (n = 4,641)	72	44,178	1.63	0.85 (0.67, 1.08)	0.179
	***P* for trend**					0.506
**Age ≥65 years**	Without (n = 65,885)	3,486	407,138	8.56	1 (reference)	-
	With (n = 18,791)	856	120,762	7.09	0.84 (0.78, 0.90)	<0.001
	**Cumulative days**					
	Nonuse (n = 65,885)	3,486	407,138	8.56	1 (reference)	-
	1–6 days (n = 10,285)	475	64,771	7.33	0.88 (0.80, 0.97)	0.008
	7–13 days (n = 4,967)	225	31,664	7.11	0.84 (0.73, 0.96)	0.009
	≥14 days (n = 3,539)	156	24,327	6.41	0.73 (0.62, 0.86)	<0.001
	***P* for trend**					<0.001

PD, Parkinson’s disease; CI, confidence interval; HR, hazard ratio; PY, person-years.

^*a*^per 1,000 person-years.

^*b*^Cox regression models were adjusted for age, sex, urbanization level, insurance amount, and comorbidities such as hypertension, diabetes mellitus, coronary artery disease, cerebrovascular diseases, head injury, depression, atrial fibrillation, liver disease, chronic infection, autoimmune disease, dementia, migraine with aura, and anemia.

## Discussion

This retrospective cohort study conducted PSM based on age, sex, and several potential confounding factors. The matching results are satisfactory, enabling the research to be comparable. However, the causal relationship between herpes zoster and PD in those aged 40 years and above remains unproven in this study, possibly because of the inclusion of a distinct population compared with prior studies and an extended follow-up period spanning 19 years. Nevertheless, a favorable correlation between herpes zoster and PD was seen in female, irrespective of the confounding variables. To our knowledge, the relationship between antiviral treatment and PD has not been extensively researched. We observed that patients who used antiviral drugs had a lower incidence of PD than those who did not (3.17 vs. 3.76 per 1,000 person-years, Table 3). After adjusting for confounding factors, the likelihood of a new PD diagnosis was 16% lower in the antiviral treatment group than in the nontreatment group. Regarding the relationship between the cumulative number of days of antiviral drug use and PD, our study revealed a significant inverse relationship between antiviral drug therapy duration and PD incidence (*P* trend = 0.022). The stratified analysis results are likewise consistent with the preceding findings, despite the fact that some results were not statistically significant.

The pathogenesis of PD is unknown. The implications of inflammation in the context of α-synuclein pathology and antiviral drug mechanisms in PD are significant and multifaceted. Research suggests that microglia, the primary immune cells in the brain, play diverse roles in PD pathogenesis. These roles range from contributing to neuronal death through the production of inflammatory factors to interacting with α-synuclein, potentially contributing to its propagation and aggregation. Microglia can also have protective functions, such as producing neurotrophic factors. Dysfunctional phagocytosis in glial cells, possibly due to lysosomal defects imparted by PD-related mutations, may contribute to microgliosis and neuroinflammation. Extracellular α-synuclein can directly activate microglia in a mutation-specific context, with α-synuclein fibrils and mutations associated with early-onset PD inducing robust immune responses. The NLRP3 inflammasome signaling in microglia, triggered by α-synuclein, is involved in activating a pro-inflammatory state, suggesting its potential role in PD [38]. Furthermore, the involvement of adaptive immunity in PD pathogenesis is supported by evidence. Studies have shown that CD4+ and CD8+ T cells are present at higher levels in the substantia nigra pars compacta of patients with PD than in control patients. This suggests a role for these cells in the inflammatory pathogenesis associated with PD. In addition, several studies demonstrate that exposure to metals and pesticides may cause PD development [17–19].

However, the hypothesis in our study posits that the herpes zoster may induce aggregation by compromising the proteostasis of α-synuclein (based on the C-terminal domain), provoke an autoimmune reaction, and ultimately cause dopamine neuron death and PD. Given that alpha herpesviruses set up latency in the peripheral nervous system, such as the dorsal root or trigeminal ganglia [39–41], usually a long time before PD develops, these links could have pathophysiological implications that may help us learn more about how PD occurs [2]. The α-synuclein protein is the main cause of Parkinson-like neurotoxicity and on how it starts and worsens [41]. One possible reason is that Lewy bodies and Lewy neuritis are mostly made up of α-synuclein. Once these pathological alterations occur, they may be observed in other brain areas, causing problems in sensory function, autonomic nerve function, and cognitive behavior. Additionally, a cross-reactivity between α-synuclein peptides and herpesvirus peptides has been demonstrated in patients with PD [1]. Consequently, the pace of PD progression may be increased by herpes zoster, especially in susceptible individuals.

The intriguing parallels between viral infections and neurodegenerative diseases across recent literature provide a substantive context for our findings. Notably, the potential role of herpes simplex virus type 1 (HSV1) in Alzheimer’s disease (AD) pathogenesis, as suggested by Itzhaki, aligns with our observations of herpes zoster’s impact on PD risk. The herpes virus’s capacity to disrupt neuronal function and incite inflammation offers a common mechanistic thread that underlies both conditions. While Itzhaki’s work posits that the presence of HSV1 in the brain could precipitate AD, especially when coupled with genetic vulnerabilities such as the ApoE4 allele [42], our study extends this viral hypothesis to the realm of PD, underscoring a potential sex-specific risk modulation in the context of antiviral interventions.

Indeed, the findings align with our expectations, highlighting sex differences in neurodegenerative disease risks and progression. Prior research has demonstrated that women face a higher lifetime risk of AD, influenced by factors like estrogen, genetic variances (e.g., APOE ε4 allele), and socio-lifestyle aspects [43–46]. Similarly, in our study, the observed higher risk of PD in females with herpes zoster might be influenced by multiple factors including biological differences in immune response, genetic susceptibility, or even differences in the prevalence or severity of herpes zoster between sexes [47, 48]. In summary, while the statistically significant finding of increased PD risk in females with herpes zoster is intriguing, it is consistent with the broader understanding of sex differences in neurodegenerative diseases. While the findings of these studies offer valuable insights, they are not directly comparable to the findings on herpes zoster and PD due to differences in disease mechanisms, the viruses involved, and the patient populations studied. However, the common thread across these studies is the potential role of viral infections in contributing to or exacerbating neurodegenerative processes. Further research exploring the biological, genetic, or social mechanisms behind these differences would be beneficial to fully understand these observations.

In addition to these findings, the significance and complexity of inflammation in relation to α-synuclein pathology and antiviral drug actions in PD are noteworthy. Previous research has demonstrated the potential neuroprotective effects of targeting inflammation in PD. Inflammasome inhibition, for instance, has been shown to protect dopaminergic neurons from α-synuclein pathology in a model of progressive PD. This indicates that strategies aimed at modulating inflammation could be beneficial in managing PD [49, 50]. Furthermore, the reported efficacy of antiviral treatments in reducing cognitive decline in AD [51] may reflect broader neuroprotective effects that also resonate with our findings in PD. The introduction of antiviral drugs such as acyclovir, valacyclovir, and famciclovir could potentially reduce the risk of PD either by suppressing viral expression or by directly inhibiting protein aggregation. The antiviral drugs in this research can disrupt the viral nucleic acid synthesis pathway. The competitive inhibition of viral DNA polymerase by triphosphate synthesis is attributed to the phosphorylation of viral thymidine kinase and host cell enzymes. This method of action hinders the deoxyguanosine triphosphate and its impact on viral DNA polymerase. Consequently, the terminal DNA cannot form a binding interaction during viral replication, causing viral death. The administration of antiviral drugs may mitigate the pathological aggregation of proteins in PD through this particular mechanism [1], thereby diminishing the probability of disease onset. This viewpoint is supported by our findings. Our observational results revealed an inverse relationship between antiviral drug use and PD risk. Especially among certain demographics like females and older adults. Moreover, a recent study conducted on mice shown that valacyclovir effectively targeted crucial pathogenic components of AD, including neuroinflammation, β-amyloid protein levels, and cholinesterase function [52]. It may even enhance cognitive function [53]. In S2 Table, our analysis revealed that the use of valacyclovir was associated with a significant 31% reduction in PD risk (HR = 0.69; 95% CI, 0.48–0.98; *P* = 0.038). This effect was not observed with famciclovir. Interestingly, acyclovir also showed a potential protective effect with a 17% risk reduction (HR = 0.83; 95% CI, 0.77–0.88; *P* < 0.001). The results of these investigations align with previous ones. Nonetheless, the present investigation did not exclude persons who were concurrently using various antiviral drugs. Therefore, our study was unable to demonstrate a direct effect of a single antiviral component on PD. It only shows that those aged 40 and above who were prescribed patients using antiviral drugs had a decreased likelihood of developing PD. Moreover, the therapy response is influenced in a manner by sex. In our data suggests a more pronounced therapeutic benefit of antiviral drugs among women. Such sex differences necessitate a nuanced approach to treatment that considers hormonal influences, genetic predispositions, and disease-specific pathologies. In conclusion, the results of this study underscore the significance of acknowledging viral infections as a plausible risk factor for neurodegenerative disorders. Additionally, the exploration of antiviral drugs shows potential as a feasible approach for the prevention or treatment of diseases. For more in-depth and up-to-date research, accessing scientific databases and journals would provide a comprehensive understanding of these associations and their implications for clinical practice and future research directions.

Conducting a large cohort study is one of the most compelling aspects of this research. Both cohorts had their comprehensive medical records analyzed, and they were followed up on for a long period. Thus, our findings are reliable, and the possibility of selection bias was reduced as much as possible. Last, this study focused on the impact of antiviral drugs on PD, recruiting 234,730 participants aged ≥40 years.

Although patients’ disease can be identified using the insurance claims data, this study still has numerous restrictions. First, neither imaging findings nor other laboratory data, such as human herpesvirus serology data, imaging tests, and viral testing, are included. Moreover, in a research based on a claims dataset, adherence to medical advice could not be confirmed and family history of PD. Second, the NHIRD could not consider residual confounding variables such as education, heredity, and diet. Additionally, our database has not detected any instances of heavy metal poisoning (ICD-9-CM: V82.5, E866.0–E866.4). Third, the statistical quality of retrospective cohort studies is typically inferior to that of randomized trials because of the potential for biases associated with adjustments made for confounding factors. To reduce this bias, we matched patients according to age, sex, and 19 comorbidities. Fourth, we need to know how long the benefit of protection against PD lasts. Hence, further research is required. Finally, the findings are restricted by one ethnic origin. Therefore, further research from across the world is necessary to confirm the efficacy of antiviral drugs in treating PD.

## Conclusions

Herpes zoster may increase PD risk, particularly among females. However, patients treated with antiviral drugs had a lower risk of developing PD by 16% than the untreated ones. The associations suggest that PD can be prevented by using antiviral drugs. Despite some limitations, the results are likely beneficial for the clinicians. Additional research is required to investigate the underlying mechanism(s).

## Supporting information

S1 Fig Schoenfeld residual test for herpes zoster and Parkinson’s disease. (TIF)

S2 Fig Schoenfeld residual test for antiviral drug use and Parkinson’s disease. (TIF)

S3 Fig Schoenfeld residual test for antiviral drug use and Parkinson’s disease in the herpes zoster cohort. (TIF)

S4 Fig Cumulative incidence curves of Parkinson’s disease in the herpes zoster and comparison cohorts. (TIF)

S5 Fig Cumulative incidence curves of Parkinson’s disease in individuals with herpes zoster treated with and without antiviral drugs. (TIF)

S1 Table Stratified analysis of Parkinson’s disease risk in patients with herpes zoster treated with or without antiviral drugs. (DOCX)

S2 Table Patients treated with antiviral drugs of different ingredients and risk of Parkinson’s disease. (DOCX)
