# Supplementary figures and images for: Use of antiviral drugs and incidence of Parkinson’s disease in Taiwan
Source: PLoS One. 2024 May 7;19(5):e0302383. doi: 10.1371/journal.pone.0302383 (PMC11075903; doi:10.1371/journal.pone.0302383)

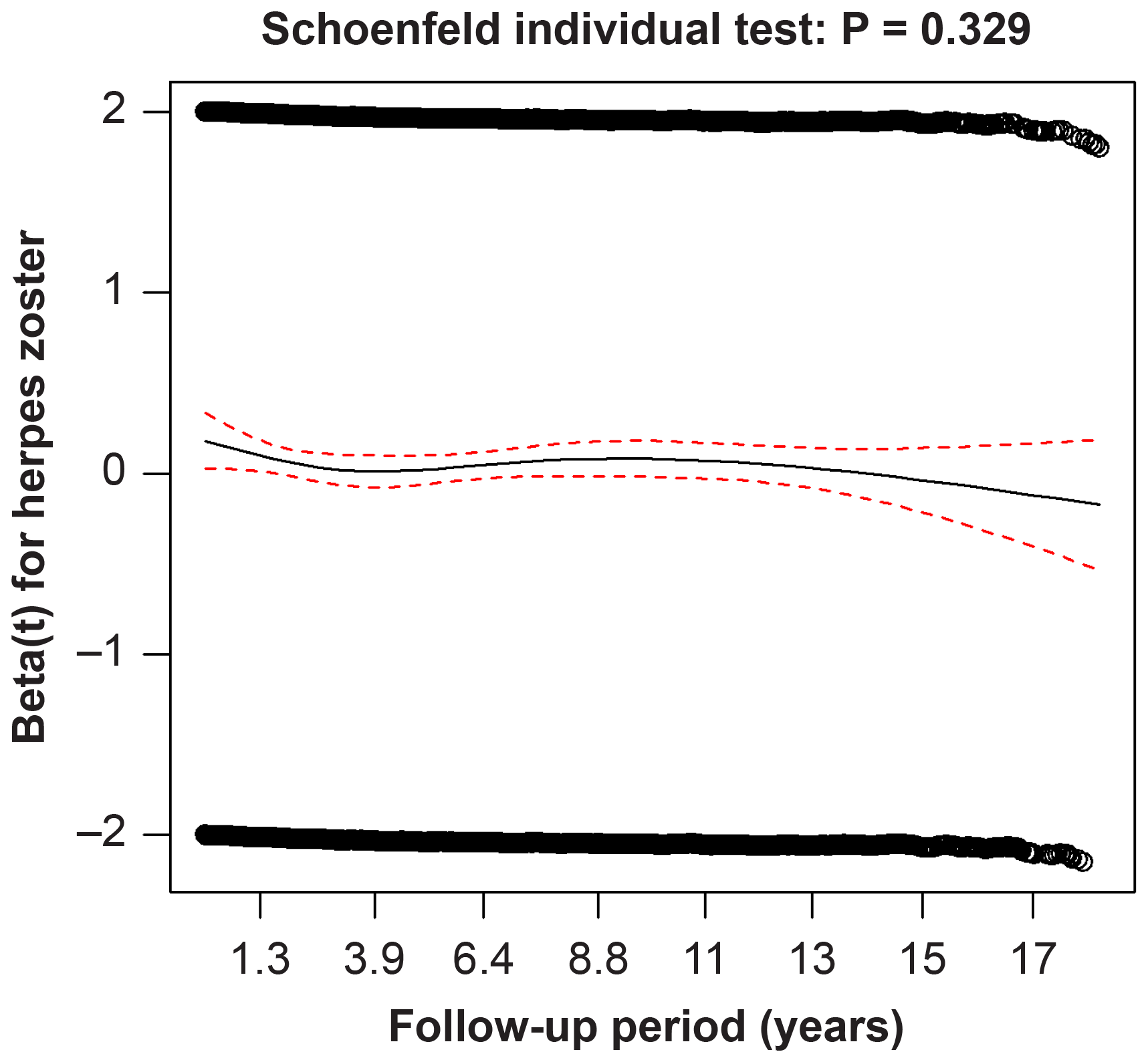

Supplement: S1 Fig — (TIF) [file pone.0302383.s001.tif]

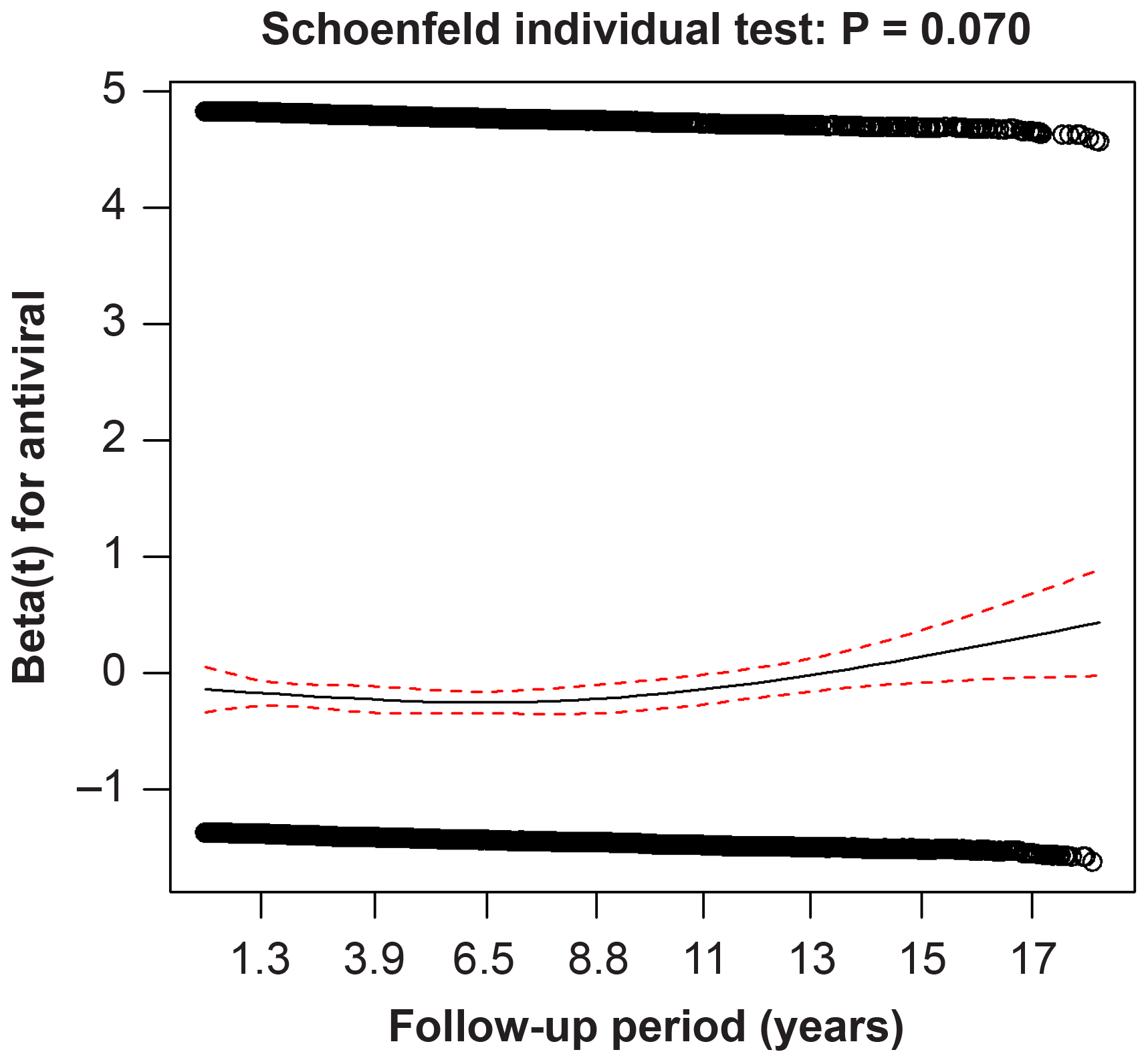

Supplement: S2 Fig — (TIF) [file pone.0302383.s002.tif]

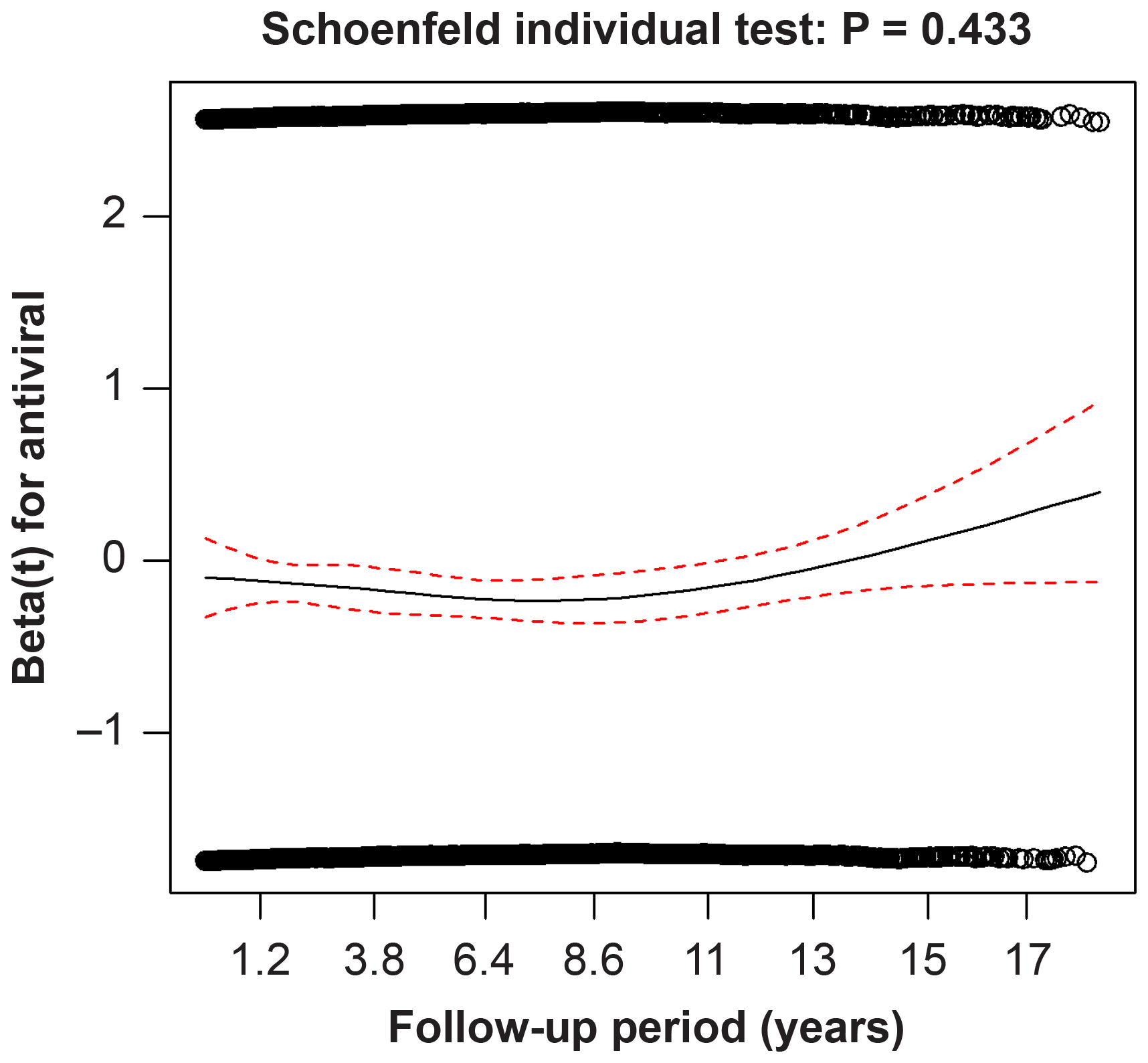

Supplement: S3 Fig — (TIF) [file pone.0302383.s003.tif]

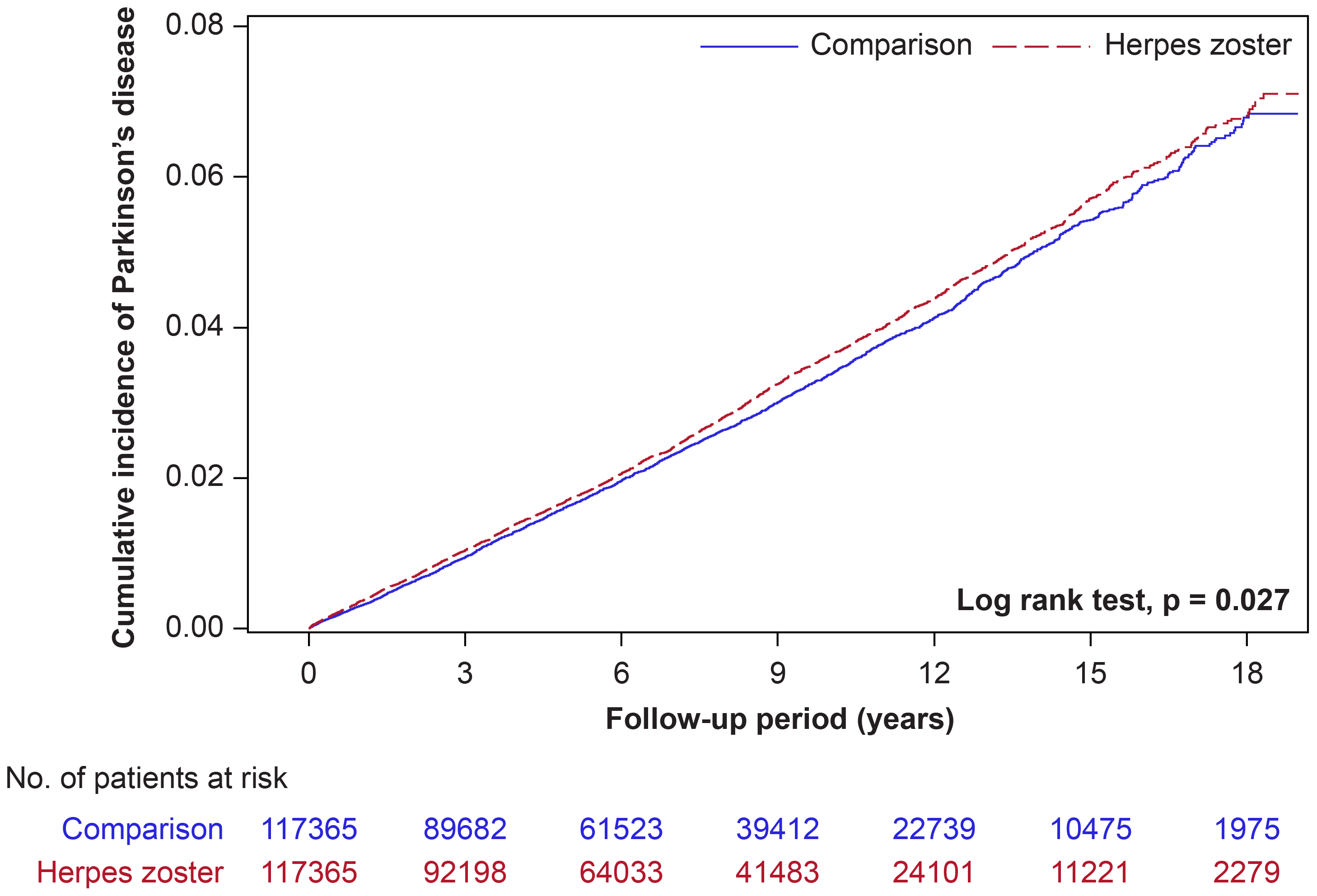

Supplement: S4 Fig — (TIF) [file pone.0302383.s004.tif]

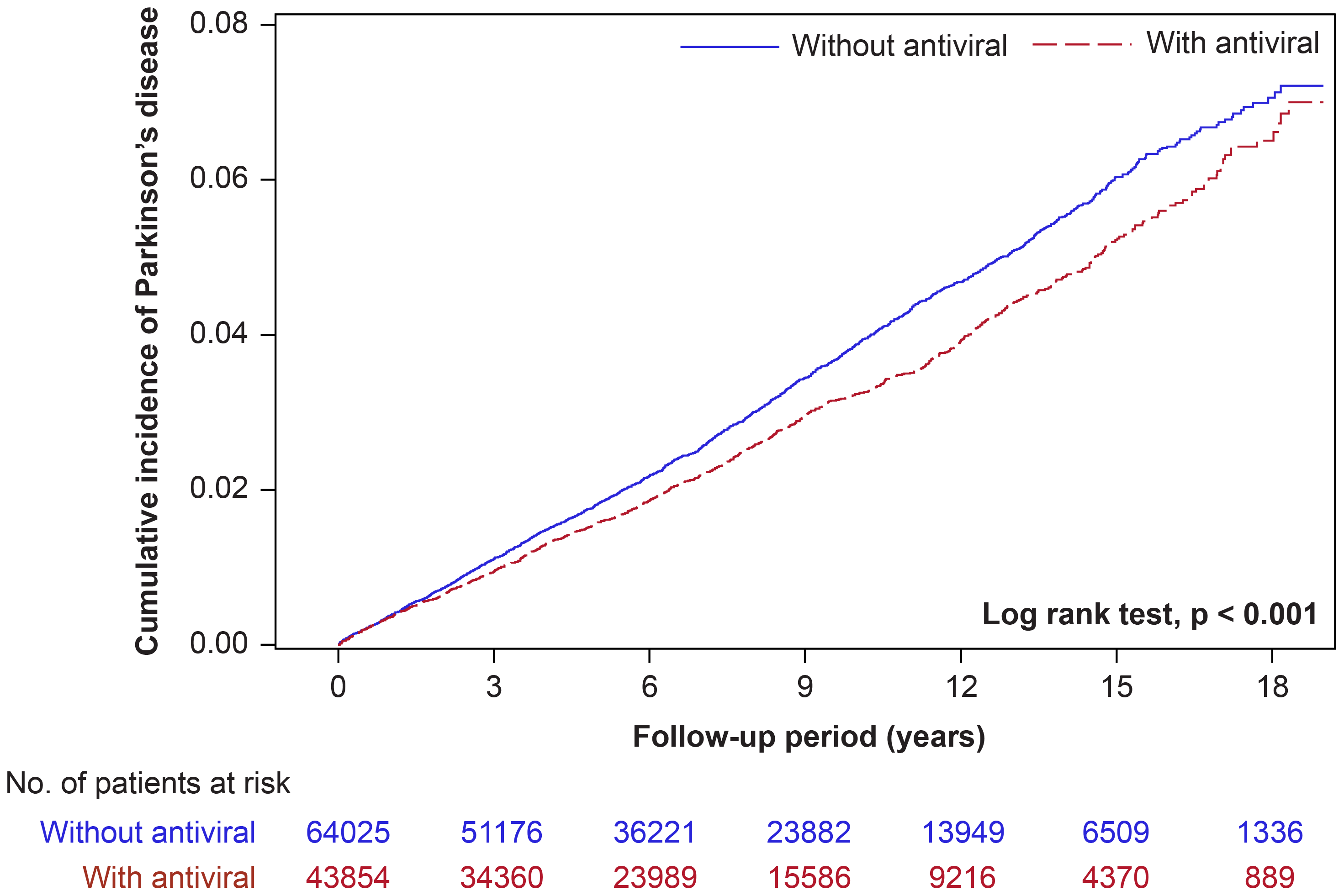

Supplement: S5 Fig — (TIF) [file pone.0302383.s005.tif]
