# Supplementary material for: Use of antiviral drugs and incidence of Parkinson’s disease in Taiwan
Source: PLoS One. 2024 May 7;19(5):e0302383. doi: 10.1371/journal.pone.0302383 (PMC11075903; doi:10.1371/journal.pone.0302383)
Supplement: S1 Table — (DOCX) [file pone.0302383.s006.docx]

**Supporting information**

**S1 Table. Stratified analysis of Parkinson's disease risk in patients with herpes zoster treated with or without antiviral drugs**

| **Population** | **Antiviral treatment** | **PD** | **PY** | **Incidence Rate*^a^*** | **Adjusted HR*^b^***  **(95% CI)** | ***P* value** |
| --- | --- | --- | --- | --- | --- | --- |
| **Total** | Without (n = 64,025) | 1,940 | 493,931 | 3.93 | 1 (reference) | - |
|  | With (n = 43,845) | 1,118 | 329,765 | 3.39 | 0.86 (0.80, 0.93) | <0.001 |
|  | **Cumulative days** |  |  |  |  |  |
|  | Non-used (n = 64,025) | 1,940 | 493,931 | 3.93 | 1 (reference) | - |
|  | 1–6 days (n = 24,916) | 604 | 183,386 | 3.29 | 0.88 (0.81, 0.97) | 0.007 |
|  | 7–13 days (n = 11,503) | 299 | 85,656 | 3.49 | 0.87 (0.77, 0.98) | 0.026 |
|  | ≥14 days (n = 7,426) | 215 | 60,724 | 3.54 | 0.80 (0.69, 0.92) | 0.002 |
|  | ***p* for trend** |  |  |  |  | 0.005 |
| **Female** | Without (n = 34,454) | 1,009 | 268,824 | 3.75 | 1 (reference) | - |
|  | With (n = 24,181) | 583 | 186,373 | 3.13 | 0.83 (0.75, 0.92) | <0.001 |
|  | **Cumulative days** |  |  |  |  |  |
|  | Non-used (n = 34,454) | 1,009 | 268,824 | 3.75 | 1 (reference) | - |
|  | 1–6 days (n = 13,843) | 330 | 104,470 | 3.16 | 0.87 (0.77, 0.98) | 0.027 |
|  | 7–13 days (n = 6,405) | 148 | 48,530 | 3.05 | 0.80 (0.67, 0.95) | 0.011 |
|  | ≥14 days (n = 3,933) | 105 | 33,373 | 3.15 | 0.77 (0.63, 0.94) | 0.010 |
|  | ***p* for trend** |  |  |  |  | 0.004 |
| **Male** | Without (n = 29,571) | 931 | 225,107 | 4.14 | 1 (reference) | - |
|  | With (n = 19,664) | 535 | 143,393 | 3.73 | 0.89 (0.8, 0.99) | 0.030 |
|  | **Cumulative days** |  |  |  |  |  |
|  | Non-used (n = 29,571) | 931 | 225,107 | 4.14 | 1 (reference) | - |
|  | 1–6 days (n = 11,073) | 274 | 78,916 | 3.47 | 0.89 (0.78, 1.02) | 0.093 |
|  | 7–13 days (n = 5,098) | 151 | 37,126 | 4.07 | 0.94 (0.79, 1.12) | 0.498 |
|  | ≥14 days (n = 3,493) | 110 | 27,351 | 4.02 | 0.82 (0.67, 1.00) | 0.048 |
|  | ***p* for trend** |  |  |  |  | 0.262 |
| **Age, 40–64 years** | Without (n = 39,800) | 505 | 333,088 | 1.52 | 1 (reference) | - |
|  | With (n = 26,647) | 318 | 222,024 | 1.43 | 0.88 (0.76, 1.01) | 0.077 |
|  | **Cumulative days** |  |  |  |  |  |
|  | Non-used (n = 39,800) | 505 | 333,088 | 1.52 | 1 (reference) | - |
|  | 1–6 days (n = 15,645) | 169 | 126,646 | 1.33 | 0.87 (0.73, 1.04) | 0.126 |
|  | 7–13 days (n = 6,858) | 83 | 56,660 | 1.46 | 0.91 (0.72, 1.15) | 0.434 |
|  | ≥14 days (n = 4,144) | 66 | 38,717 | 1.70 | 0.86 (0.67, 1.12) | 0.269 |
|  | ***p* for trend** |  |  |  |  | 0.550 |
| **Age, ≥65 years** | Without (n = 24,225) | 1,435 | 160,843 | 8.92 | 1 (reference) | - |
|  | With (n = 17,198) | 800 | 107,741 | 7.43 | 0.85 (0.78, 0.92) | <0.001 |
|  | **Cumulative days** |  |  |  |  |  |
|  | Non-used (n = 24,225) | 1,435 | 160,843 | 8.92 | 1 (reference) | - |
|  | 1–6 days (n = 9,271) | 435 | 56,739 | 7.67 | 0.88 (0.79, 0.98) | 0.023 |
|  | 7–13 days (n = 4,645) | 216 | 28,995 | 7.45 | 0.85 (0.74, 0.98) | 0.024 |
|  | ≥14 days (n = 3,282) | 149 | 22,006 | 6.77 | 0.75 (0.63, 0.89) | <0.001 |
|  | ***p* for trend** |  |  |  |  | <0.001 |

PD, Parkinson’s disease; CI, confidence interval; HR, hazard ratio; PY, person-years.

*^a^*per 1,000 person-years.

*^b^*Cox regression models were adjusted for age, sex, urbanization level, insurance amount, and comorbidities such as hypertension, diabetes mellitus, coronary artery disease, cerebrovascular diseases, head injury, depression, atrial fibrillation, liver disease, chronic infection, autoimmune disease, dementia, migraine with aura, and anemia.
