# Supplementary material for: Use of antiviral drugs and incidence of Parkinson’s disease in Taiwan
Source: PLoS One. 2024 May 7;19(5):e0302383. doi: 10.1371/journal.pone.0302383 (PMC11075903; doi:10.1371/journal.pone.0302383)
Supplement: S2 Table — (DOCX) [file pone.0302383.s007.docx]

S2 Table. Patients treated with antiviral drugs of different ingredients and risk of Parkinson's disease

| **Antiviral treatment** | **Group** | **PD** | **PY** | **Incidence Rate***^a^* | **Adjusted HR***^b^*  **(95% CI)** | ***P* value** |
| --- | --- | --- | --- | --- | --- | --- |
| Acyclovir | Without (n=175,550) | 4,859 | 1,289,774 | 3.77 | 1 (reference) | - |
|  | With (n=45,070) | 1,116 | 360,662 | 3.09 | 0.83(0.77, 0.88) | <0.001 |
| Valacyclovir | Without (n=217,952) | 5,944 | 1,638,085 | 3.63 | 1 (reference) | - |
|  | With (n=2,668) | 31 | 12,351 | 2.51 | 0.69(0.48, 0.98) | 0.038 |
| Famciclovir | Without (n=216,747) | 5,881 | 1,626,034 | 3.62 | 1 (reference) | - |
|  | With (n=3,873) | 94 | 24,402 | 3.85 | 0.97(0.79, 1.20) | 0.807 |

PD, Parkinson’s disease; CI, confidence interval; HR, hazard ratio; PY, person-years.

*^a^*per 1,000 person-years.

*^b^*Cox regression models were adjusted for age, sex, urbanization level, insurance amount, and comorbidities such as hypertension, diabetes mellitus, coronary artery disease, cerebrovascular diseases, head injury, depression, atrial fibrillation, liver disease, chronic infection, autoimmune disease, dementia, migraine with aura, and anemia.
